# A systematic review of neoadjuvant and definitive immunotherapy in locally advanced head and neck squamous cell carcinoma

**DOI:** 10.1002/cam4.5815

**Published:** 2023-03-19

**Authors:** Udit Nindra, Joshua Hurwitz, Dion Forstner, Venessa Chin, Richard Gallagher, Jia Liu

**Affiliations:** ^1^ Department of Medical Oncology Liverpool Hospital Sydney New South Wales Australia; ^2^ Department of Medical Oncology Campbelltown Hospital Sydney New South Wales Australia; ^3^ The Kinghorn Cancer Centre St Vincent's Hospital Darlinghurst New South Wales Australia; ^4^ The University of New South Wales Kensington New South Wales Australia; ^5^ GenesisCare Darlinghurst New South Wales Australia; ^6^ The Garvan Institute of Research Camperdown New South Wales Australia; ^7^ The University of Sydney Camperdown New South Wales Australia

**Keywords:** curative, definitive immunoradiotherapy, head and neck cancer, immunotherapy, neoadjuvant chemoimmunotherapy

## Abstract

**Background:**

Patients with locally advanced head and neck squamous cell carcinoma (HNSCC) require multi‐modality treatment. Immune checkpoint inhibitors (ICIs) are now standard of care in management of recurrent/metastatic HNSCC. However, its role in the definitive and neoadjuvant setting remains unclear.

**Methods:**

A literature search was conducted that included all articles investigating ICI in untreated locally advanced (LA) HNSCC. Data was extracted and summarised and rated for quality using the Cochrane risk of bias tool.

**Results:**

Of 1086 records, 29 met the final inclusion criteria. In both concurrent and neoadjuvant settings, the addition of ICI was safe and did not delay surgery or reduce chemoradiotherapy completion. In the concurrent setting, although ICI use demonstrates objective responses in all published trials, there has not yet been published data to with PFS or OS benefit. In the neoadjuvant setting, combination ICI resulted in superior major pathological response rates compared to ICI monotherapy without a significant increase adverse event profiles, but its value in improving survival is not clear. ICI efficacy appears to be affected by tumour characteristics, in particular PD‐L1 combined positive score, HPV status and the tumour microenvironment.

**Conclusions:**

There is significant heterogeneity of ICI use in untreated LA HNSCC with multiple definitive concurrent and neoadjuvant protocols used. Resultantly, conclusions regarding the survival benefits of adding ICI to standard‐of‐care regimens cannot be made. Further trials and translational studies are required to elucidate optimal ICI sequencing in the definitive setting as well as better define populations more suited for neoadjuvant protocols.

## 1 INTRODUCTION

Head and neck cancer accounts for over 700,000 new cases of cancer globally and 350,000 deaths annually.^1^ Most patients present with locally advanced disease that requires multimodality treatment. Standard treatment protocols are typically surgery alone, surgery and adjuvant therapy, primary radiotherapy or concurrent chemoradiation. Despite treatment advances, locoregional recurrence occurs in up to 50% of patients, another factor contributing to HNSCC‐related deaths. High rates of short‐term morbidity related to the use of chemotherapy such as mucositis, xerostomia, haematological toxicity, nausea and acute kidney injury are ongoing challenges.^2^

Locally advanced HNSCC often involve multiple sites and subsites of the upper aerodigestive tract with regional metastatic disease. Traditional surgical approaches to HNSCC can result in significant cosmetic and functional morbidity. Sequential technological advances and their application to surgery have resulted in less morbid operative approaches and surgical outcomes. This has been particularly highlighted by the increasing number of human papilloma virus (HPV) ‐driven oropharyngeal SCC which led to the development of transoral robotic surgery (TORS), now an accepted part of the treatment paradigm. However, patients who have extensive nodal involvement with extra‐nodal extension or incomplete resections often need adjuvant treatment. Thus there is significant room for neoadjuvant immune checkpoint inhibitors (ICI) to possibly reduce the need for adjuvant therapy.^3^

There is growing evidence for neoadjuvant ICI in multiple malignancies. This approach has demonstrated a high pathological response rate impacting relapse‐free survival rates in tumour streams such as melanoma, colorectal cancer, breast cancer and lung cancer.^4^, ^5^, ^6^ These studies demonstrated that a higher pathological complete response is associated with more extended event‐free survival in patients treated with neoadjuvant chemotherapy and ICI versus chemotherapy alone. In HNSCC, pathological complete response (pCR) is an excellent surrogate and immediate biomarker for better long term response.^7^

Several other systematic reviews on neoadjuvant ICI in HNSCC have been reported;^8^, ^9^ however, they are lacking both quality and risk of bias assessment of reported studies. Furthermore, recently presented and published trials have evolved the neoadjuvant and definitive ICI space in locally advanced HNSCC. Our systematic review aims to differentiate with regards to our synthesis of multiple up‐to‐date trials and abstracts identified along with the quality assessment of these trials, looking for bias and heterogeneity.

## 2 METHODS

In September 2022, a literature search was conducted across five databases – Medline, PubMed, Cochrane Library, Embase and Google Scholar. Two authors (UN and JH) independently used the multiple combinations of search terms: “immunotherapy”, “immune checkpoint inhibitors”, “pembrolizumab”, “avelumab”, “nivolumab”, “head and neck cancer” and “squamous” to include all relevant articles to the topic. Our search strategy was as follows: “Head and neck cancer” AND (“immunotherapy” or “immune checkpoint inhibitors” OR “pembrolizumab” OR “avelumab” OR “nivolumab” OR “durvalumab”) AND (“definitive” OR “neoadjuvant”).

**FIGURE 1:**
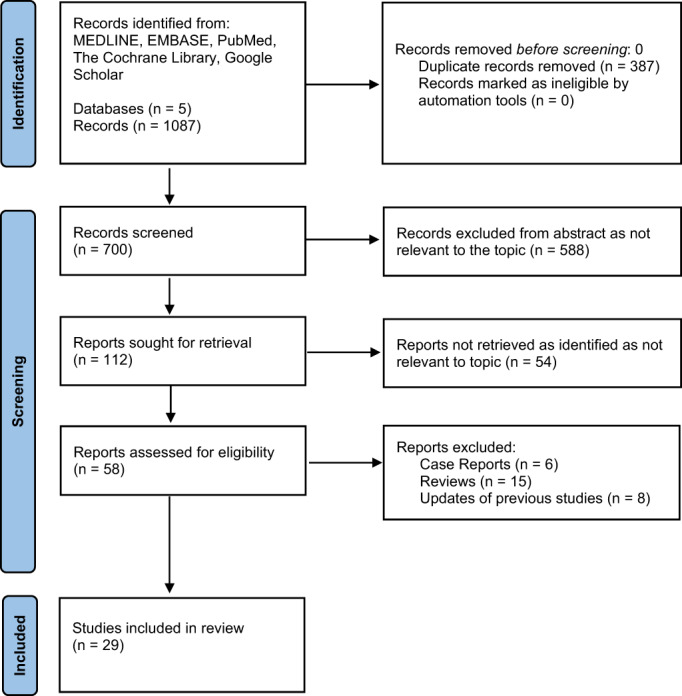
PRISMA diagram for literature review of neoadjuvant or definitive immunotherapy in head and neck squamous cell carcinoma.

Our search included all published trials in English to date including early‐stage phase 1 single‐arm studies and abstracts. Our preliminary search identified 1083 articles from which 387 duplicates were removed and 696 were screened. In total, 588 abstracts were removed due to lack of relevance to the topic and 108 articles were retrieved for full review (Figure 1). Review articles, case reports and clinical updates were excluded.

Study quality was assessed using the ROBINS‐I tool from the Cochrane Library^10^ and the modified Oxford Centre for Evidence Based‐Medicine Score. The ROBINS‐I tool evaluated bias in seven domains: confounding, selection, classification of the intervention, deviation from the intervention, missing data, measurement of outcome and selection of report results. Each domain was assessed for the risk of bias and the overall risk of bias was determined as a cumulative result of all domains.

## 3 RESULTS

Overall, 29 articles (number of patients = 2730) were included in the final analysis (Figure 1). We stratified the articles into five subgroups. This included definitive immunotherapy with concurrent chemoradiotherapy, definitive immunotherapy with concurrent radiotherapy alone, neoadjuvant immunotherapy plus radiotherapy alone, neoadjuvant immunotherapy plus chemotherapy alone and finally neoadjuvant immunotherapy alone prior to surgery. Data regarding treatment protocols, planned primary and secondary outcomes, and published results were extracted and summarised in Table 1. Of the 29 studies, 12 investigated ICI in the definitive setting, while 17 looked into neoadjuvant ICI.

**TABLE 1:** Summary of studies investigating ICIs in locally advanced HNSCC.

Study, Author, Year	Study phase	Study protocol	Study population	Number of patients	Primary outcomes	Secondary outcomes	Results
Trials investigating definitive immunotherapy with chemoradiotherapy							
NCT02764593 Gillison et al. 2019^14^	I	Arm 1: Nivolumab + weekly cisplatin + RT Arm 2: Nivolumab + 3 weekly cisplatin + RT Arm 3: Nivolumab + Cetuximab + RT Arm 4: Nivolumab + RT alone	HPV +ve stage III–IVB disease	40; 10 per arm	DLT; AE	Progression or death events	Arm 1: 0/8 DLT, 0/10 events Arm 2: 0/8 DLT, 1/9 events Arm 3: 1/8 DLT (mucositis), 1/10 events Arm 4: 2/8 DLT (lipase, mucositis, fatigue), 3/10 events
NCT02586207 Powell et al. 2020^12^	I	Pembrolizumab + Cisplatin + RT	Cisplatin eligible HPV +ve or HPV −ve stage III–IVB disease	59	AE	CRR on Day 100	8.8% patients discontinued pembrolizumab due to AE; 98.3% completed RT without significant delay (>5 days); End of treatment CRR was 85.3% and 78.3% for HPV +ve and HPV ‐ve disease, respectively
NCT02952586 (Javelin‐100) Lee et al. 2021^11^	III	Arm 1: Avelumab + Cisplatin + RT Arm 2: Cisplatin + RT alone	HPV +ve or HPV −ve stage III–IVB disease	Total: 697 Arm 1: 350 Arm 2: 347	PFS	OS, pCR rate, time to locoregional failure, ORR, DOR, AE, HRQOL	Median PFS not reached in either Avelumab + CRT arm or CRT alone arm; Primary objective not met
NCT03426657 (CheckRad‐CD8) Hecht et al. 2021^13^, ^15^	II	Cisplatin + Docetaxel + Durvalumab + Tremilimumab + RT	HPV +ve or HPV −ve stage III–IVB disease	79	DLT; CD8 T cell density	PFS, pCR Rate; OS	1‐year PFS 79%; 2‐year PFS 73%; 1‐year OS 89%; 2‐year OS 86%. AE ≥grade 3 in 95% and mainly consisted of dysphagia (53%), leucopenia (48%) and infections (29%). DLT mainly consisted of hepatitis (10%)
NCT03426657 (CheckRad‐CD8) Hecht et al. 2021^13^, ^15^	II	Cisplatin + Docetaxel + Durvalumab + Tremilimumab + RT	HPV +ve or HPV −ve stage III–IVB disease	79	DLT; CD8 T cell density	PFS, pCR Rate; OS	In the expansion cohort, neither increase of tremelimumab dosage nor its omission affected pathologic response to induction chemo‐immunotherapy with cisplatin/docetaxel/durvalumab
NCT02777385 Clump et al. 2022^16^	II	Arm 1: Cisplatin + RT + Pembrolizumab Arm 2: Cisplatin + RT followed by Pembrolizumab	HPV +ve or HPV −ve stage III–IVB disease	Total: 80 Arm 1: 41 Arm 2: 39	DLT; 1‐year local failure rate	PFS	The 2‐year PFS for sequential pembro of 89% versus 78% for concurrent pembro. OS at 2 years was 94% for sequential and 78% for concurrent pembro. Median PFS and OS was NR
NCT03040999 (Keynote 412) Michaels et al. 2022^17^	III	Arm 1: Pembrolizumab + Cisplatin + RT Arm 2: Placebo + Cisplatin + RT	HPV +ve or HPV −ve stage III–IVB disease	Total: 804 Arm 1: 402 Arm 2: 402	EFS	OS, Safety	Non‐statistically significant association between pembro and EFS (24 month EFS 63.2% vs. 56.2%). Outcomes associated with PD‐L1 CPS. No difference in OS between two arms
Trials investigating definitive immunotherapy with radiotherapy							
NCT02707588 (PembroRad) Bourhis et al. 2020^18^	II	Arm 1: Pembrolizumab + RT Arm 2: Cetuximab + RT	Cisplatin ineligible HPV +ve or HPV −ve stage III–IVB disease	Total: 131 Arm 1: 66 Arm 2: 65	Locoregional control	PFS, OS, AE, HRQOL	Grade ≥3 AE 74% in Pembro arm v 92% in Cetux arm; LRC at 15 m 89% in Cetux arm vs. 60% in Pembro arm. No difference in PFS at 25 m. 2‐pCR OS 55% in Cetux arm vs. 62% in Pembro arm (HR 0.83; *p* = 0.49)
NCT03162731 Johnson et al. 2020^19^	I	Ipilimumab + Nivolumab + RT	HPV +ve or HPV −ve stage IVA–IVB disease	24	Safety	1‐year PFS, 1‐year OS	Grade ≥3 AE in 71% of patients; no grade ≥4 AE occurred. At median follow‐up 16 months, 87.5% of patients were alive. LRC was 100%
NCT02609503 Weiss et al. 2020^33^	II	Pembrolizumab + RT	Cisplatin ineligible HPV +ve or HPV −ve stage III–IVB disease	29	PFS	OS, AE, ORR, CRR, LRC, HRQOL	With median follow‐up of 21 months, estimated 24 months PFS and OS were 71% and 75%, respectively. Toxicities were in line with those expectant of RT; of note 58.6% had ≥grade 3 lymphopenia
NCT02999087 (REACH) Tao et al. 2020^20^	III	Arm 1: Cisplatin + RT Arm 2: Cetuximab + Avelumab + RT Arm 3: Cetuximab + RT	HPV +ve or HPV −ve stage III–IVB disease	Total: 82 Arm 1: 21 Arm 2: 41 Arm 3: 20	PFS	OS, AE	The most common AE were radiation dermatitis, mucositis and dysphagia. Grade ≥4 AE occurred in 5/41 patients in the experimental arms. PFS and OS data not currently available
NCT03258554 (NRG‐HN004) Mell et al. 2019^34^	III	Durvalumab + RT	Cisplatin ineligible HPV +ve or HPV −ve stage III–IVB disease	10	DLT, PFS, OS	LRC, AE, HRQOL, p16 status	In 10 patients recruited to date, no DLT or grade ≥4 toxicities were observed. No grade ≥3 toxicities to durvalumab were noted either. All 10 patients completed definitive RT
Trials investigating neoadjuvant immunotherapy with radiotherapy							
NCT03238365 Luginbuhl et al. 2022^22^	I	Arm 1: Nivolumab + RT followed by surgery Arm 2: Nivolumab + Tadalafil + RT followed by surgery	Any stage HPV +ve or HPV −ve resectable HNSCC	Total: 46 Arm 1: 20 Arm 2: 25	Change in immune cell polarisation	Prevalence of intertumoral immune cell populations	Neoadjuvant treatment was well tolerated with no grade 3 or greater AE. 25/45 (54%) had a pathological treatment response (pTR) with 7% demonstrating complete response
NCT03247712 Leidner et al. 2021^21^	I	three doses neoadjuvant Nivolumab + RT followed by surgery then three doses of adjuvant Nivolumab	HPV +ve or HPV −ve stage III–IVB disease	21	Surgical delay	Tumour size regression	No surgical delay resulted with neoadjuvant treatment. Major pathological response and pCR occurred in 86% and 67%, respectively. Clinical to pathological downstaging occurred in 90% of patients
Trials investigating neoadjuvant immunotherapy with chemotherapy							
NCT03342911 Zinner et al. 2020^23^	II	Nivolumab + Paclitaxel + Carboplatin	Stage III–IV HPV −ve or stage II–III HPV +ve disease	27	pCR	MPR	All 27 patients had surgery following neoadjuvant protocol. 11/26 (42%) had pCR at the primary site. 9/23 (39%) HPV negative patients had pCR. 26/27 patients alive at 16 months
NCT03107182 Rosenberg et al. 2021^24^	II	Nivolumab + Nab‐Paclitaxel + Carboplatin followed by either TORS or definitive RT or definitive CRT	HPV +ve stage III–IVB disease	73	DRR	AE, PFS, OS, LRC	DRR following neoadjuvant treatment was 70.8%. De‐escalated treatment was administered in 84.9%. 2‐year OS for full cohort was 90.4%. Grade 4 toxicity was <10% in all treatment arms
Trials investigating neoadjuvant immunotherapy with surgery							
NCT03003637 Vos et al. 2021^35^	II	Arm 1: Nivolumab Arm 2: Nivolumab + Ipilimumab	Stage II–IV HPV +ve or HPV −ve resectable HNSCC	Total: 32 Arm 1: 6 Arm 2: 26	Surgical delay, MPR	AE	MPR occurred in 35% in Nivo/Ipi arm compared with 17% in Nivo alone arm. There were no surgical delays in either arm. No patients with MPR had recurrence during 24 months median follow‐up
NCT02488759 Ferris et al. 2021^25^	II	Nivolumab	Any stage HPV +ve or HPV −ve resectable HNSCC	52	AE, Surgical delay	Radiographic response, PPR	Any grade AE occurred in 73.1% with grade ≥3 occurring in 15.4%. No surgical delays were noted. No patient had pCR. 4/17 HPV positive patients and 1/17 HPV negative patient showed PPR
NCT02919683 Schoenfeld et al. 2020^26^	II	Arm 1: Nivolumab Arm 2: Nivolumab + Ipilimumab	Untreated OPSCC (≥T2, or clinically node positive)	Total: 29 Arm 1: 14 Arm 2: 15	Safety, Volumetric response	Pathological response, ORR, PFS, OS	There were no surgical delays. There was evidence of response in both the N and N + I arms (volumetric response 50%, 53%; pathologic downstaging 53%, 69%; pathologic response 54%, 73%, respectively)
NCT02296684 Uppaluri et al. 2020^28^	II	Single dose pembrolizumab followed by surgery and post‐operative CRT; high risk patients received adjuvant pembrolizumab	HPV −ve, stage III–IV resectable HNSCC	36	LRC, Pathological treatment effect	AE, Distant metastases rate	No surgical delay. pTR of >50% occurred in 22% while pTR of >10% occurred in 44%. 16.7% 1‐year relapse in high‐risk population. PPR correlated with PD‐L1, immune infiltrate and IFN‐gamma activity
NCT02296684 Uppaluri et al. 2021^29^	II	2 doses of pembrolizumab followed by surgery and post‐operative CRT; high risk patients received adjuvant pembrolizumab	HPV −ve, stage III–IV resectable HNSCC	29	LRC, Pathological treatment effect	AE, Distant metastases rate	No surgical delay. pTR of >50% occurred in 44% of patients. No new safety signals compared with earlier study
NCT02641093 Wise‐Draper et al. 2021^31^	II	Pembrolizumab followed by surgery and adjuvant CRT + Pembrolizumab (7 total doses)	HPV −ve T3‐T4 and/or ≥2 nodal metastases or clinical ENE	76	AE, DFS	Tumour immune response	1‐year DFS was 67% in high‐risk group versus 93% in intermediate risk group. 32 patients had PPR with 6 MPR. PD‐L1 expression correlated with MPR and PPR (*p* < 0.01). Grade ≥3 AE in 62% of patients
NCT03144778 Ferrarotto et al. 2020^36^	I	Arm 1: Two cycles Durvalumab pre‐operatively Arm 2: Two cycles Durvalumab + tremelimumab pre‐operatively	Stage II–IV HPV +ve or HPV −ve resectable OPSCC	Total: 28 Arm 1: 14 Arm 2: 14	Tumour immune response	AE, ORR	CD8 density 1.31 in D arm while 1.15 in D + T arm. 43% in each arm had clinical response. 29% had MPR in the total cohort. 14% had grade ≥3 AE. At median follow‐up of 15.8 months, all patients were alive
NCT03618654 Curry et al. 2021^37^	I	Arm 1: 1x Durvalumab pre‐operatively Arm 2: 1x Durvalumab with Metformin daily in preoperative setting	Any stage HPV +ve or HPV −ve resectable HNSCC	Total: 38 Arm 1: 9 Arm 2: 29	Immune cell polarisation	IHC marker alteration; tumour size	Pathological effect at the primary site was seen in 55% in the total cohort, 37.5% in Arm 1 and 60% in Arm 2 (*p* = 0.4). Changes in TME not currently available
NCT03575598 Bernal et al. 2020^38^	I	Nivolumab + Sitravatinib	HPV +ve or HPV −ve T2‐4a, N0‐2 or T1 > 1 cm‐N2 OPSCC	10	Tumour immune response	AE, PPR	At 69 weeks all patients alive with no recurrence. Best responders had higher percentage of PD‐L1 tumour‐associated macrophages at baseline
NCT03737968 Hong et al. 2021^27^	II	Arm 1: Single dose Durvalumab pre‐operatively Arm 2: Single dose Durvalumab + tremelimumab pre‐operatively	Stage II–IV HPV +ve or HPV −ve resectable HNSCC	Total: 44 Arm 1: 20 Arm 2:24	LRR, Distant metastases rate	LRC, DMFS, PFS	Neoadjuvant D or D + T had acceptable safety profiles and was not associated with surgical delay. Tumour shrinkage observed in 70.5%; MPR achieved in three patients. LRC was 95.5%
NCT03021993 Knochelmann et al. 2021^30^	II	3–4 bi‐weekly Nivolumab pre‐operatively	Stage II to IV HPV +ve or HPV −ve resectable OPSCC	12	ORR	OS	ORR was 33%. With a median follow‐up of 2.23 years, 10/12 patients remained alive. There were no surgical delays or significant AE signals
NCT04393506 Zhong et al. 2021^32^	I	Camrelizumab + Apatinib pre‐operatively followed by CRT	Stage III to IVa HPV +ve or HPV −ve resectable OPSCC	21	MPR	2‐year OS, 2‐year PFS	No toxicity of grade ≥3 occurred. 1/21 patients experienced surgical delay. MPR rate was 40% including one patient with pCR. All four patients with PD‐L1 CPS ≥20 had MPR
NCT03854032 Luginbuhl et al. 2022^39^	II	Arm 1: Nivolumab + BMS986205 pre‐operatively Arm 2: Nivolumab alone pre‐operatively	Stage II–IV HPV +ve or HPV −ve resectable HNSCC	Total: 42 Arm 1: 31 Arm 2: 11	ORR	PPR, Immune cell polarisation	The addition of IDO‐inhibitor to anti‐PD1 did not result in a significant increase in radiographic or pathologic response over nivolumab alone

Abbreviations: AE, adverse events; CRR, complete response rate; DLT, dose‐limiting toxicity; DMFS, distant metastases‐free survival; DOR, duration of response; DRR, deep response rate; EFS, eventfree survival; ENE, extra‐nodal extension; HNSCC, head and neck squamous cell carcinoma; HPV, human papillomavirus; HRQOL, health‐related quality of life; ICIs, Immune checkpoint inhibitors; IHC, immunohistochemistry; LA HNSCC, Locally advanced head and neck squamous cell carcinoma; LRC, locoregional control; LRR, locoregional relapse rate; MPR, major pathological response; OPSCC, oropharyngeal SCC; ORR, objective response rate; pCR, pathological complete response; PFS, progression‐free survival; PPR, partial pathological response; RT, radiotherapy; TME, tumour microenvironment.

### 3.1 Definitive Immunotherapy and Chemoradiotherapy

Seven published trials investigated immunotherapy in the definitive setting combined with CRT.^11^, ^12^, ^13^, ^14^, ^15^, ^16^, ^17^ Discontinuation rates or dose‐limiting toxicity (DLT) events were low and observed in <10% of patients. The Javelin‐100 trial is one of two phase III trials in the definitive ICI + CRT space, comparing avelumab with standard of care CRT versus standard of care CRT alone.^11^ With a median follow‐up of 14.5 months, this trial failed to meet its primary endpoint of improved PFS, but demonstrated similar safety signals compared with the phase I and II studies with serious adverse events occurring in 36% in the avelumab arm compared with 32% in the CRT alone arm. In this trial's post‐hoc analysis, PD‐L1 expression appeared to be a predictive biomarker of survival outcomes with greater benefit seen in PD‐L1 TPS ≥25%. Recently, the primary results of the Keynote‐412 study were reported in which pembrolizumab with concurrent chemoradiation was compared with chemoradiation alone.^17^ Although the study did trend towards improved event‐free survival (EFS), this result was not statistically significant and no difference in OS between the two arms was noted. PD‐L1 CPS, similar to the Javelin‐100 trial, appeared to act as a predictive biomarker with those patients with ≥20 PD‐L1 CPS potentially having greater ICI efficacy.^17^

### 3.2 Definitive Immunotherapy with Radiotherapy

Definitive immunotherapy with radiotherapy alone has been investigated in five trials, with safety being the most common primary endpoint. Only one trial, PembroRad by Bourhis et al,^18^ has published results comparing definitive ICI‐RT with standard of care treatment. Although grade three or more AE were less in the ICI‐RT arm compared with Cetuximab‐RT, the study did not demonstrate PFS survival differences between the two arms in a cisplatin‐ineligible population. OS results favoured the ICI group but did not reach statistical significance (HR 0.83, *p* = 0.49). Locoregional control (LRC) appeared to be better in the Cetuximab‐RT arm than ICI‐RT (89% vs. 60% at 15 months). LRC was high at 100% at 16 months with the use of dual ICI (Ipilimumab and Nivolumab) in conjunction with RT in a small single‐arm Phase I trial.^19^ The REACH and NRG‐HN004 studies are the two phase III trials with preliminary results published to date.^20^ However, overall survival data, the primary objective of both studies, is still pending.

### 3.3 Neoadjuvant Immunotherapy and Radiotherapy

In the neoadjuvant setting, immunotherapy has been used in combination with radiation alone to explore pathological response rates and surgical delay. Currently, there are two published studies in this space. Leidner et al. investigated three doses of neoadjuvant nivolumab with RT followed by surgery and three doses of adjuvant nivolumab.^21^ This protocol did not lead to surgical delay. Major pathological response (>50% tumour regression) was observed in 14/21 (86%) of cases. Luginbuhl et al. investigated tadalafil, a phosphodiesterase‐5 inhibitor, in combination with nivolumab and RT in the neoadjuvant setting.^22^ They demonstrated that pathological treatment response (≥20% tumour regression) to this combination occurred in 54% of patients, with 7% showing a complete response. Currently, there is no published data on the overall or recurrence free survival benefit of neoadjuvant ICI with RT compared with definitive standard of care regimens.

### 3.4 Neoadjuvant Immunotherapy and Chemotherapy

Concurrent ICI with chemotherapy in the neoadjuvant HNSCC setting has been investigated in two published trials. The first was conducted by Zinner et al. where combination nivolumab, paclitaxel and carboplatin were used in a mostly HPV‐negative LA HNSCC cohort.^23^ In this study, all 27 patients had definitive surgery post neoadjuvant treatment with 42% demonstrating pCR as defined by less than 10% residual tumour at time of resection. Rosenberg et al. conducted a Phase II study in 73 patients with HPV positive disease only, investigating the role of de‐escalation therapy depending on pathological tumour response to neoadjuvant chemoimmunotherapy.^24^ This study used combination nivolumab, nab‐paclitaxel and carboplatin with the primary outcome being deep response rate (DRR) which they defined by 10% or less residual tumour. They demonstrated that following neoadjuvant treatment, 70.8% had DRR, which resulted in de‐escalation of adjuvant therapy in 84.9% of cases. Using this model, the cohort's 2‐year OS was 93.3%, concluding that neoadjuvant chemoimmunotherapy provides significant pathological response, allowing for de‐escalation of adjuvant therapy in the LA HNSCC setting.

### 3.5 Neoadjuvant Immunotherapy

Neoadjuvant ICI has been investigated in 13 published trials. Nivolumab monotherapy was investigated by Ferris et al. in both the HPV positive and negative setting.^25^ Although no surgical delays were observed and AE profiles were considered safe, rates of partial pathological response were low, seen in 4/17 HPV positive and 1/17 HPV negative tumours.^25^ Vos et al. investigated combination ICI compared with single‐agent nivolumab demonstrating combination therapy improved MPR rates (35% vs. 17%). Those patients with MPR had excellent disease control, and no recurrence was observed in 24 months median follow‐up. AE profile of combination therapy was within predicted immune‐related toxicity. Combination ipilimumab and nivolumab was also investigated by Schoenfield et al. in untreated oral cavity SCC.^26^ Evidence of response was identified with both single agent nivolumab and combination treatment (volumetric response 50%, 53%; pathologic downstaging 53%, 69%; RECIST response 13%, 38%; and pathologic response 54%, 73%, respectively). Three patients had complete response in combination treatment compared to one with nivolumab alone. After 14.2 months of median follow‐up, 1‐year progression‐free survival was 85% and overall survival was 89%.^26^ Combination ICI with durvalumab and tremelimumab is also being investigated by Hong et al.^27^ In early results from their study of 44 patients, neoadjuvant durvalumab or durvalumab plus tremelimumab had acceptable safety profiles and was not associated with surgical delay. Tumour shrinkage was observed in 70.5% of patients with MPR achieved in three patients. Locoregional control was 95.5%. Comparative results between the durvalumab and durvalumab plus tremelimumab arms are not currently available.

In the HPV‐negative setting, Uppaluri et al investigated the role of single‐dose neoadjuvant pembrolizumab followed by surgery and adjuvant ICI and chemoradiotherapy.^28^ Partial pathological response was observed in 44% of patients with neoadjuvant pembrolizumab and 22% demonstrating at least 50% tumour regression. No significant adverse event signals were raised, nor surgical delay. In this cohort, PD‐L1 expression appeared to correlate with pathological tumour response (*p* = 0.02). Uppaluri followed this initial study with a similar protocol but with two doses of neoadjuvant pembrolizumab followed by surgery,^29^ which doubled the rate of 50% tumour regression to 44%. In terms of increasing ICI pre‐operative doses, Knochelmann et al. investigated using 3–4 bi‐weekly nivolumab doses pre‐operatively^30^ whereby patients who did not have RECIST 1.1 progression after three cycles of neoadjuvant nivolumab received a fourth cycle prior to surgery. They demonstrated an ORR (as defined by defined as pathologic complete response + pathologic partial response) of 33%, with 10 of 12 patients in the study alive at a median follow‐up of 2.2 years.

Wise‐Draper et al also investigated the benefit of neoadjuvant pembrolizumab followed by surgery, adjuvant CRT and adjuvant pembrolizumab to a total of seven cycles.^31^ They found that 32/76 patients had at least partial pathological response (PPR) to neoadjuvant ICI. Similar to Uppaluri's study, Wise‐Draper found that PPR correlated with PD‐L1 with those with CPS ≥1 demonstrated increased response to neoadjuvant ICI.^31^ Zhong et al also demonstrated that PD‐L1 combined positive score (CPS) is suggestive of MPR.^32^ They investigated 21 patients with camrelizumab plus apatinib pre‐operatively followed by CRT. MPR occurred in all four patients with PD‐L1 CPS ≥20.

### 3.6 Quality of studies

**FIGURE 2:**
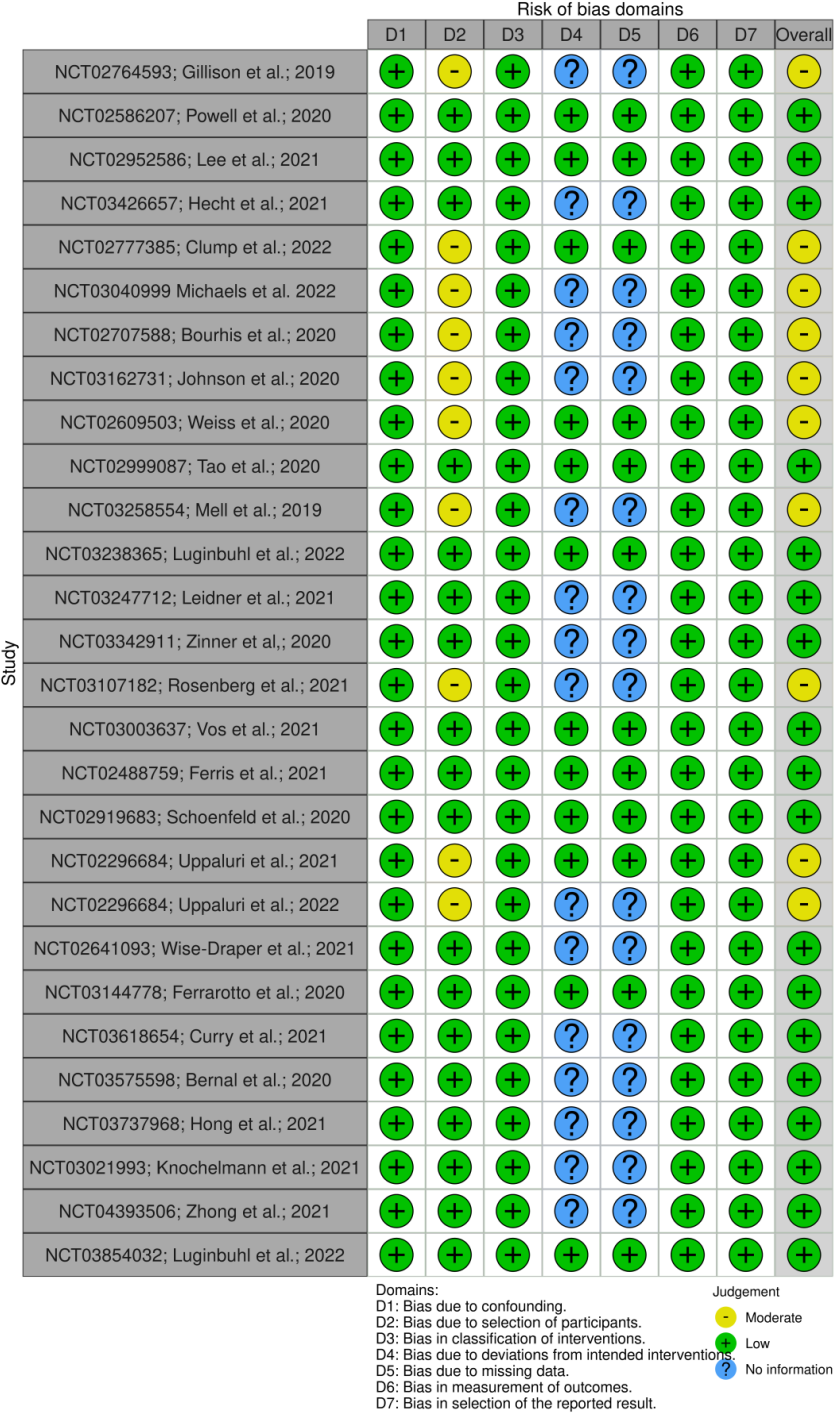
Quality assessment of studies based on Cochrane ROBINS‐I tool for evidence‐based medicine.

Among the 29 studies included, nine demonstrated a moderate risk of bias, while no study was found to have any serious or critical risks. The primary domain influencing bias was selection. Of these 29 studies, 13 unique inclusion criteria were used, resulting in significant heterogeneity in study populations and a unified understanding of results. Furthermore, 16 out of 29 trials are in abstract form alone. In these abstracts, no information could be elicited regarding bias resulting from deviation from planned intervention or bias resulting from missing data. However, no evidence of bias from these domains was found in the remaining 13 articles. The overall bias assessment has been summarised in Figure 2.

## 4 DISCUSSION

As demonstrated, multiple researchers have investigated the role of immunotherapy in the neoadjuvant and definitive setting for treatment of locally advanced HNSCC and this is a rapidly evolving field. Our paper provides a comprehensive overview of all currently published trials. Published studies show that adding immunotherapy to definitive chemoradiation or radiation alone does not appear to increase toxicity risks and can potentially increase locoregional control. For patients with PD‐L1 expressing tumours and those with p16 positivity, immune blockade is likely to improve pathological response further. However, significant work is needed to standardise treatment protocols (including the quality of radiotherapy), how response to treatment is defined (including uniform definitions of pathological responses assessment) and populations where immunotherapy are most likely to be of benefit.

Overall, the LA HNSCC space has significant heterogeneity regarding study populations and treatment protocols in published trials. Investigators have stratified inclusion criteria by HPV status, cisplatin eligibility and stage of disease, complicating the ability to provide recommendations on ICI usage in the neoadjuvant and definitive setting. In the definitive space, five different ICI have been trialled with two phase III studies, JAVELIN‐100 and Keynote‐412, currently published. One other current phase III study with preliminary toxicity results is the REACH study which is an ongoing investigation of the benefit of avelumab with either radiotherapy or cetuximab plus radiotherapy. PFS or OS data is not currently available for this study. Given these results, ICI use in the definitive setting lacks strong evidence to support its routine use compared with standard of care treatment. This recommendation may change pending the results of the REACH study.^20^ Although these trials are disappointingly negative, the reasoning behind this is currently not known. One possible theory is that ablative radiation to all draining lymph nodes potentially may be negating the effect of ICIs as primed T cells are subsequently killed, so strategies to sequence ICIs better in definitive setting, or tailor radiation dose and field is needed in future trials.^33^

In the neoadjuvant setting, a recent meta‐analysis has demonstrated that in resectable HNSCC, neoadjuvant immunotherapy has favourable outcomes without surgical delay or increased toxicity.^34^ Our study adds to this by providing the most up‐to‐date review of the literature, highlighting the heterogeneity of study protocols and potential bias that could influence results. We also formally assessed for safety and tolerability in HNSCC and reported pathological response rates. Pathological response in the heterogeneous population of HNSCC has been found to range between 17 and 52% with a significant proportion of patients having pathological down staging allowing for de‐escalated adjuvant regiments.^8^, ^25^ These response rates are seen in both HPV positive and HPV negative disease.^25^ While combination immunotherapy is promising, no phase III studies have been published comparing ICI use with standard of care treatment, and survival data is eagerly awaited.

Patient selection is of significant importance in ICI use in LA HNSCC and predictive biomarkers are required to rationale ICI use in the neoadjuvant and definitive setting. HPV positive patients have increased pathological response to ICI in the recurrent/metastatic HNSCC setting and early signals from Zinner and Powell are also suggestive of this.^12^, ^23^ Powell et al. is currently the only study in the definitive ICI + CRT setting to investigate the influence of HPV on the efficacy of treatment. They demonstrated that end of treatment complete response rate, defined at 150 days post CRT completion, was higher in the HPV positive subgroup at 85.3% versus 78.3% in the HPV negative subgroup.^12^ JAVELIN‐100 provided HPV status of patients included in the trial, pCR, ORR, PFS and OS data subgroup of HPV positive versus HPV negative patients was not included. Thus, further research into the importance of HPV status in the LA HNSCC is required. Another pertinent question is whether ICI should be reserved for those patients who are cisplatin ineligible. Powell, Bourhis, Weiss and Mell all included only cisplatin ineligible patients in their studies with no significant increased safety signals in what would be expected to be a frailer cohort of patients.^12^, ^18^, ^35^, ^36^

Several trials questioned the timing of treatment and the amount of necessary immunotherapy to provide a response. Clump et al. demonstrated that sequential rather than concurrent ICI provided an improved 2‐year OS benefit of 94% versus 78% in the definitive space.^16^ Median PFS and OS was not reached in either cohort. This may provide early signals that timing of ICI therapy in the definitive setting could influence outcomes.

On the other hand, Uppaluri conducted two trials investigating additional neoadjuvant ICI cycles demonstrating that two cycles of pembrolizumab compared with one increased pathological tumour response of >50% from 22% to 44%.^28^, ^29^ However, whether this translates to a PFS or OS benefit is unknown.

Another important consideration is the heterogeneity of result reporting affecting bias in result interpretation in the neoadjuvant setting. Major pathological response (MPR) has been traditionally defined as viable residual tumour of less than or equal to 10% and is often used as a surrogate end point for survival in patients with multiple other solid organ malignancies such as lung cancer.^37^ Vos, Wise‐Draper, Zhong and Ferrarotto defined MPR as viable tumour of less than or equal to 10%.^31^, ^32^, ^38^, ^39^ However, some studies, such as those by Uppaluri et al. did not report MPR but instead reported pathological treatment response of either ≥10% or ≥50% in their cohorts.^28^, ^29^ Similarly, Rosenberg et al. used a surrogate marker of deep response rate (DRR) to define a ≥50% tumour regression post neoadjuvant treatment.^24^ These differences in reporting pathological response post‐neoadjuvant treatment need to be considered when interpreting the efficacy of therapy. An international consensus working group to standardise definitions of pathological response in all head and neck studies involving immunotherapy is urgently required, to allow uniformity in data synthesis and outcome definitions in clinical trials moving forwards.

Lastly, the tumour microenvironment response is envisaged to play a role in the efficacy of neoadjuvant immunotherapy. Multiple trials have investigated T‐cell populations in patients with increased response to treatment.^28^, ^39^ Ferrarotto et al. investigated the role CD8+ T cell density as a marker of prognosis by treating 28 patients with neoadjuvant ICI.^40^ They found that neoadjuvant that CD8+ T cell density was higher post neoadjuvant ICI which trended towards higher likelihood of MPR.^40^ To adapt the tumour microenvironment to generate improved response to ICI, Curry et al. investigated the role of neoadjuvant durvalumab with metformin.^41^ Their study has demonstrated increased pathological response with combination treatment but without statistical significance (60% vs. 37.5%, *p* = 0.4).^41^ To further explore the influence of the tumour microenvironment on ICI outcomes in the neoadjuvant setting, Bernal et al. investigated adding in sitravatinib, a multi‐target tyrosine kinase inhibitor, with nivolumab pre‐operatively.^42^ They theorised that increased density of M1‐type tumour‐associated macrophages and decreased incidence of myeloid‐derived suppressor cells from this combination would amplify treatment response. In 10 patients they demonstrated a greater disease response in those with a higher percentage of PD‐L1 positive tumour‐associated macrophages.^42^ Whether this combination alone leads to survival improvements is still being investigated. Recently, indoleamine 2,3‐dioxygenase (IDO) inhibitors have also been trialled, without success, with neoadjuvant immunotherapy in untreated LA HNSCC to improve pathological response.^43^

## 5 CONCLUSION

There is a wide array of ICI use in untreated LA HNSCC with multiple definitive and neoadjuvant protocols with and without chemotherapy, radiation therapy, targeted therapy and metabolic drugs. As a result, definitive conclusions regarding ideal treatment modalities still require further research. Currently, in the definitive setting, although ICI use demonstrates objective responses in all trials, two large phase III study failed to show PFS benefit compared with standard of care CRT.^11^ In the neoadjuvant setting, phase III trials have not been published. It is becoming more evident that ICI in LA HNSCC is affected by tumour characteristics, specifically PD‐L1 CPS, as well as the tumour microenvironment. Multiple trials have investigated tumour microenvironment modulation's role in increasing ICI response and activity. HPV status also appears to play a role in ICI responsiveness, but the extent to which it affects clinical outcomes remains unclear. Future trials in a biomarker‐selected population with optimised sequencing of IO, particularly in conjunction with radiotherapy which ablates draining lymph nodes are required.

## AUTHOR CONTRIBUTIONS

**Udit Nindra:** Conceptualization (equal); data curation (equal); formal analysis (equal); investigation (equal); methodology (equal); writing – original draft (equal); writing – review and editing (equal). **Joshua Hurwitz:** Conceptualization (equal); data curation (equal); formal analysis (equal); investigation (equal); methodology (equal); writing – original draft (equal); writing – review and editing (equal). **Dion Forstner:** Conceptualization (equal); supervision (equal); validation (equal); writing – review and editing (equal). **Venessa Chin:** Conceptualization (equal); supervision (equal); writing – review and editing (equal). **Richard Gallagher:** Conceptualization (equal); supervision (equal); writing – review and editing (equal). **Jia Liu:** Conceptualization (equal); supervision (equal); writing – original draft (equal); writing – review and editing (equal).

## FUNDING INFORMATION

This research did not receive any specific grant from funding agencies in the public, commercial or not‐for‐profit sectors.

## CONFLICT OF INTEREST STATEMENT

The authors have no conflict of interest to declare.

## Data Availability

The datasets for this manuscript are not publicly available but requests to access the datasets should be directed to Udit Nindra (udit.nindra@health.nsw.gov.au).
